# Supplementary material for: Modeling glioblastoma heterogeneity as a dynamic network of cell states
Source: Mol Syst Biol. 2021 Sep 16;17(9):e10105. doi: 10.15252/msb.202010105 (PMC8444284; doi:10.15252/msb.202010105)
Supplement: Supplementary file 5 — Source Data for Figure 3 [file MSB-17-e10105-s001.zip › Figure3A_sourcedata/GSEA_3065/nosigdb_state6.GseaPreranked.1622718443993/IVY_CTMVP.html]

Details for gene set IVY\_CTMVP[GSEA]

|  || Dataset | state6 |
| Phenotype | NoPhenotypeAvailable |
| Upregulated in class | na\_neg |
| GeneSet | IVY\_CTMVP |
| Enrichment Score (ES) | -0.6840156 |
| Normalized Enrichment Score (NES) | -2.327367 |
| Nominal p-value | 0.0 |
| FDR q-value | 0.0 |
| FWER p-Value | 0.0 |
Table: GSEA Results Summary

  

Fig 1: Enrichment plot: IVY\_CTMVP      
 Profile of the Running ES Score & Positions of GeneSet Members on the Rank Ordered List

  

| PROBE | GENE SYMBOL | GENE\_TITLE | RANK IN GENE LIST | RANK METRIC SCORE | RUNNING ES | CORE ENRICHMENT || 1 | FILIP1L |  |  | 175 | 0.183 | 0.0161 | No |
| 2 | ARHGAP29 |  |  | 453 | 0.128 | 0.0095 | No |
| 3 | SVIL |  |  | 471 | 0.126 | 0.0324 | No |
| 4 | LBH |  |  | 695 | 0.106 | 0.0278 | No |
| 5 | STC1 |  |  | 1133 | 0.079 | -0.0067 | No |
| 6 | OLFML2A |  |  | 1756 | 0.055 | -0.0670 | No |
| 7 | TGFBI |  |  | 2809 | 0.026 | -0.1820 | No |
| 8 | FN1 |  |  | 2839 | 0.026 | -0.1802 | No |
| 9 | STEAP1B |  |  | 3851 | 0.004 | -0.2950 | No |
| 10 | TPM2 |  |  | 4007 | 0.001 | -0.3126 | No |
| 11 | PHACTR2 |  |  | 4052 | -0.000 | -0.3176 | No |
| 12 | KCNJ2 |  |  | 4147 | -0.002 | -0.3279 | No |
| 13 | TAGLN |  |  | 5323 | -0.025 | -0.4572 | No |
| 14 | PRDM1 |  |  | 5349 | -0.025 | -0.4551 | No |
| 15 | PDLIM1 |  |  | 5379 | -0.026 | -0.4533 | No |
| 16 | CCDC102B |  |  | 5482 | -0.028 | -0.4595 | No |
| 17 | UACA |  |  | 5586 | -0.030 | -0.4653 | No |
| 18 | THBS1 |  |  | 5670 | -0.032 | -0.4685 | No |
| 19 | F2R |  |  | 5881 | -0.036 | -0.4855 | No |
| 20 | FRZB |  |  | 6536 | -0.051 | -0.5502 | No |
| 21 | ARHGAP18 |  |  | 6628 | -0.054 | -0.5501 | No |
| 22 | TGFBR2 |  |  | 6727 | -0.057 | -0.5502 | No |
| 23 | IGFBP7 |  |  | 7318 | -0.075 | -0.6028 | No |
| 24 | GNG11 |  |  | 7504 | -0.082 | -0.6079 | No |
| 25 | NID1 |  |  | 7561 | -0.084 | -0.5978 | No |
| 26 | KDELR3 |  |  | 8317 | -0.134 | -0.6577 | Yes |
| 27 | COL18A1 |  |  | 8322 | -0.134 | -0.6317 | Yes |
| 28 | FSTL1 |  |  | 8373 | -0.143 | -0.6093 | Yes |
| 29 | TM4SF1 |  |  | 8392 | -0.146 | -0.5827 | Yes |
| 30 | LAMA4 |  |  | 8483 | -0.157 | -0.5621 | Yes |
| 31 | COL5A2 |  |  | 8514 | -0.165 | -0.5332 | Yes |
| 32 | COL4A2 |  |  | 8525 | -0.168 | -0.5014 | Yes |
| 33 | LAMC1 |  |  | 8618 | -0.199 | -0.4728 | Yes |
| 34 | PCOLCE |  |  | 8639 | -0.209 | -0.4342 | Yes |
| 35 | LAMB1 |  |  | 8640 | -0.209 | -0.3932 | Yes |
| 36 | SERPINH1 |  |  | 8646 | -0.212 | -0.3521 | Yes |
| 37 | COL4A1 |  |  | 8661 | -0.218 | -0.3109 | Yes |
| 38 | PRSS23 |  |  | 8688 | -0.233 | -0.2681 | Yes |
| 39 | PXDN |  |  | 8708 | -0.244 | -0.2223 | Yes |
| 40 | CTGF |  |  | 8715 | -0.254 | -0.1730 | Yes |
| 41 | MCAM |  |  | 8727 | -0.266 | -0.1219 | Yes |
| 42 | GPX8 |  |  | 8771 | -0.324 | -0.0631 | Yes |
| 43 | ITGB1 |  |  | 8777 | -0.335 | 0.0022 | Yes |
Table: GSEA details [plain text format]

  

Fig 2: IVY\_CTMVP: Random ES distribution      
 Gene set null distribution of ES for **IVY\_CTMVP**
